# The effectiveness of artificial intelligence health education accurately linking system on self-management in non-specific lower back pain patients

**DOI:** 10.3389/fpubh.2025.1630329

**Published:** 2025-09-11

**Authors:** Yun-Hua Li, Na Li, Zhi-xia Liu, Shuang Du, Yujiang Shuai, Renjun Yang, Lei Xu, Xiaoyan Li, Yang Jiang, Wenwen Li

**Affiliations:** ^1^School of Education, Chengdu College of Arts and Sciences, Chengdu, China; ^2^Department of Rehabilitation, Jintang First People's Hospital, Chengdu, China; ^3^School of Nursing, Henan University of Science and Technology, Luoyang, China; ^4^Jitang College, North China University of Science and Technology, Tangshan, China

**Keywords:** artificial intelligence, AI-HEALS, large language model, non-specific lowerback pain, mobile health, RCT

## Abstract

**Background:**

Integrating artificial intelligence (AI) with mobile health is revolutionizing chronic disease management. Non-specific lower back pain (NSLBP), a leading worldwide disabling condition, negatively impairs patient quality of life and psychological status. Standard treatments, mostly pharmacological and physiotherapies, do not offer long-term support for self-management. Consequently, we developed the AI-Health Education Accurately Linking System (AI-HEALS) to investigate its application in improving self-management, alleviating pain, and enhancing overall life quality for NSLBP patients.

**Methods:**

This study utilizes a randomized controlled trial (RCT) to evaluate the effectiveness of a three-month AI-HEALS intervention in improving self-management among patients with NSLBP. Participants are randomly assigned to either a control group receiving standard care or an intervention group receiving standard care supplemented by the AI-HEALS program. The intervention features an AI-powered, voice-activated interactive Q&A system, along with physiological monitoring, regular reminders, and tailored educational content. These services are primarily delivered via a WeChat official account titled “NSLBP Health Management Expert.” The AI-HEALS system builds its knowledge base based on NSLBP treatment guidelines to ensure the accuracy and reliability of the information provided. The primary outcome measure is pain intensity, while secondary outcomes assess self-management behaviors, psychological well-being, and physiological parameters.

**Discussion:**

AI-HEALS program combines AI with mobile health to provide an organized platform for efficient home care of NSLBP, alleviating pain, enhancing quality of life, and lessening dependency upon conventional medical resources. Results from this study will establish AI-HEALS’ effectiveness in managing chronic diseases and provide a science basis for subsequent health intervention.

**Clinical trial registration:**

Identifier, CHICTR2400090707.

## 1 Introduction

Non-specific lower back pain (NSLBP) refers to a condition caused by acute or chronic damage to the muscles, fascia, and other soft tissues of the lower back due to trauma, lumbar disk herniation, and other factors (1, 2). It is a common global health issue that significantly impacts people’s quality of life and work efficiency (1, 3). The prevalence of NSLBP globally is approximately 7.6%, with a rapid increase observed over the past two decades (4). Compared to spinal injuries or other diseases with clear physiological etiologies, the treatment and prognosis of NSLBP face greater challenges due to its often unclear causes (1, 2). The condition not only leads to long-term and recurrent pain in patients but may also cause functional impairments and activity limitations, increasing the risk of anxiety and depression (5, 6). These symptoms significantly reduce the quality of life, including sleep disturbances and reduced social activities (3, 7, 8). The persistent nature of chronic pain not only exacerbates the psychological and economic burden on family members but also affects emotional communication and social functions within the family (9, 10). Moreover, patients’ increased dependency on external support necessitates additional care and support from family members, potentially leading to an imbalance in family dynamics (9, 10).

From a social and economic perspective, NSLBP is one of the leading causes of work absenteeism and decreased work capacity (11–13). Studies indicate that NSLBP, along with widespread lower back pain, is the leading cause of work disability among the working-age population (11–13). This condition not only imposes a significant economic burden on individuals and families but also exerts substantial pressure on the socioeconomic system, resulting in extensive consumption of medical resources and notable productivity losses (11–13). According to data from the Global Burden of Disease (GBD) Study, low back pain ranked as the sixth leading cause of disability-adjusted life years (DALYs) worldwide in 2019, accounting for approximately 63.7 million DALYs—an increase of nearly 47% since 1990 (14). Furthermore, the 2021 GBD Study confirmed that low back pain remains among the top 10 causes of DALYs globally, underscoring its persistent and considerable public health impact (15).

Current conventional interventions for NSLBP encompass a variety of approaches, including pharmacotherapy, physical therapy, psychological interventions, and lifestyle modifications (3, 7, 16). Pharmacological treatments primarily rely on non-steroidal anti-inflammatory drugs and muscle relaxants, which can effectively alleviate pain but may cause gastrointestinal issues and dependency when used long-term (17, 18). Physical therapies, such as massage, heat therapy, and acupuncture, improve local blood circulation and relieve muscle tension and pain, yet they typically require patients to regularly visit healthcare facilities, increasing both time and financial burdens (19–21). Psychological interventions, such as cognitive-behavioral therapy, aim to alter patients’ perceptions and coping strategies regarding pain, helping to mitigate the psychological burdens associated with chronic pain (22, 23). However, such psychological interventions require the involvement of professional psychologists, making them difficult to widely implement in resource-limited areas (22, 23). As for lifestyle adjustments, such as increasing moderate exercise and improving sleep quality, while these have long-term benefits for symptom and pain management, they also depend heavily on the patient’s self-management capabilities and sustained motivation, which can result in significant variability in effectiveness (24–26).

On the whole, there are some limitations with existing treatments for NSLBP. To begin with, some of these treatments necessitate patients’ multiple visits to health facilities, which can be inconvenient for patients residing in rural areas or with tight workloads. Secondly, these traditional treatments such as pharmacotherapy and physical therapy carry adverse effects and may not be applicable to all patients. Thirdly, the unequal availability of resources for psychological intervention restricts these types of intervention from being used over wider areas.

Currently, over 75% of the global population has access to mobile phones with internet capabilities, while more than 57% of households have an internet connection (27, 28). In Europe, these figures reach as high as 99 and 86%, respectively (27, 28). These statistics highlight the indispensable role of smartphones in daily life and their potential significance in health management and lifestyle adjustments, particularly in facilitating communication between healthcare professionals and patients. Traditionally, mobile health interventions primarily relied on voice services or text-based Short Message Service (29–31). However, with the increasing availability and usability of applications, there has been a significant rise in the number of smartphone apps specifically designed to alter health behaviors (29, 32). These apps encompass a wide range of areas, including pain management, chronic disease management, weight control, and the management of smoking and other addictive substances (29–32). Taking the study by Piette et al. as an example, they designed an intervention combining artificial intelligence with an interactive voice response system for 278 veterans suffering from chronic back pain over a 10-week period (33). The results indicated that this intervention was as effective as cognitive behavioral therapy for chronic pain (CBT-CP) delivered by therapists over the phone, but required significantly less therapist time. Such AI-assisted CBT-CP interventions could enable the same number of therapists to effectively serve a larger number of patients. Economically, the study by Fatoye also yielded positive results. Implementing remote multidisciplinary management for NSLBP patients, they found that remote rehabilitation could save approximately 16,000 Naira (about 44.3 USD) per patient (34). These observations highlight the cost savings from telemedical treatment using advanced communication media and their potential for extensive use.

In this context, the deployment of the Artificial Intelligence-Health Education Accurately Linking System (AI-HEALS) via WeChat, China’s largest social media site, assumes special relevance. It employs cheap, ubiquitous mobile communication technology so that patients are able to receive support and management from their own homes. AI-HEALS intervention content relies on the HAPA-MTM theoretical model, addressing not only physiological symptom management but also highlighting psychological health and changes in behavior, providing an integrated and extended approach to disease management (35–37). Highlight of the system includes AI-based knowledge Q&A from a tailored knowledge base, tracking of physiological markers and behaviors, different reminder services, and message dissemination tailored to each individual. Such an innovative intervention model is well-suited for contemporary society’s needs and can potentially provide efficient support and management for many patients with NSLBP.

In summary, this study is intended to assess the efficacy of AI-HEALS intervention program among patients with NSLBP on their self-management outcome by conducting a randomized controlled trial (RCT) in the following areas:

- Evaluate the efficacy of the three-month multifaceted AI-HEALS intervention in enhancing physiological indicators among the NSLBP population;
- Assess the efficacy of the three-month integrated AI-HEALS intervention for improving psychological indicators in the NSLBP population;
- Evaluate the efficacy of the three-month overall AI-HEALS intervention in enhancing behaviors of self-management among NSLBP individuals.

## 2 Materials and methods

### 2.1 Design

**Figure 1:**
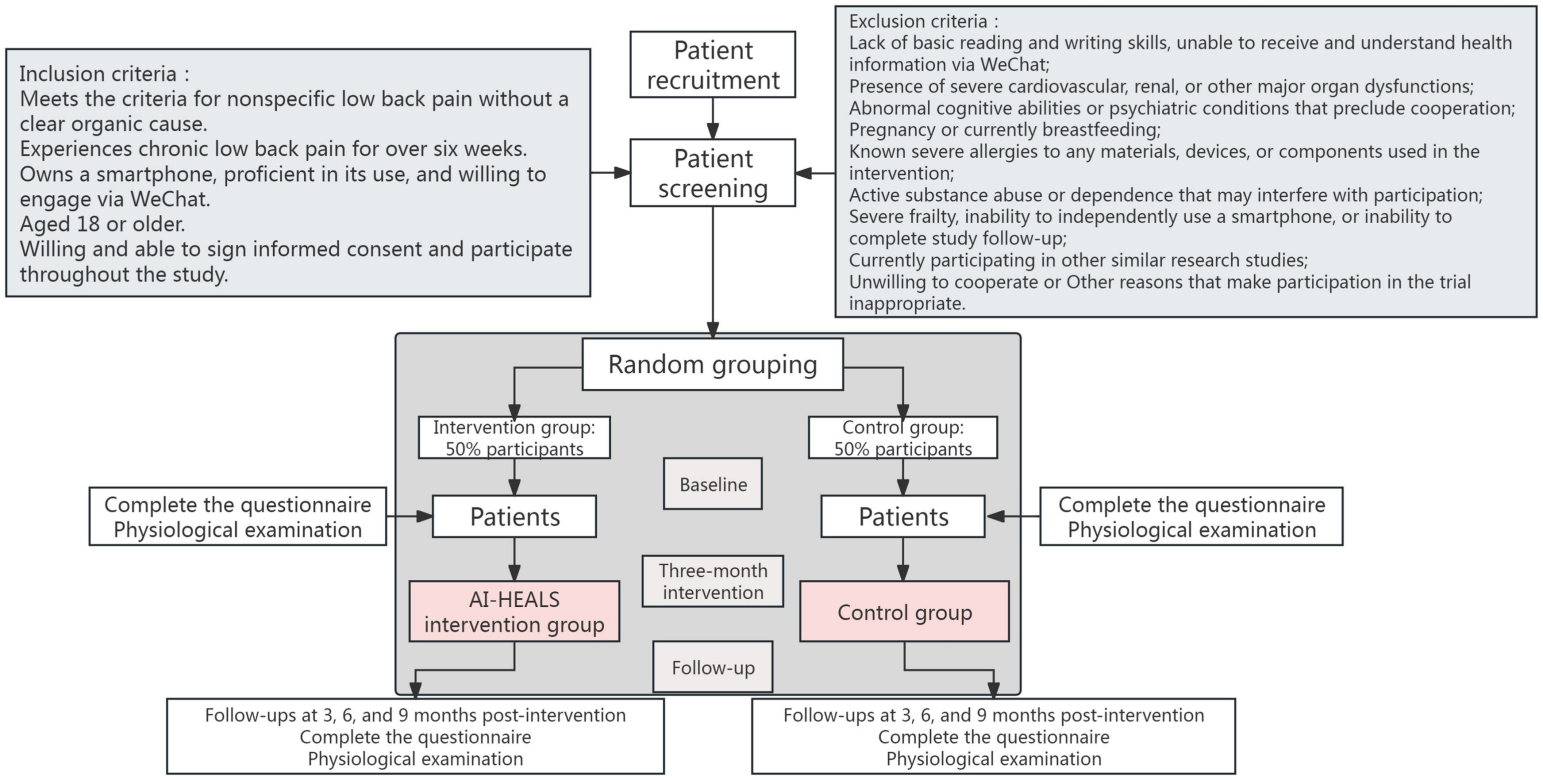
Flow chart of patient recruitment and study implementation.

This is a mixed-method study utilizing a randomized, single-blind, parallel-group controlled clinical trial conducted at a public comprehensive hospital in Chengdu, Sichuan, China. The study involves individuals diagnosed with NSLBP and is a single-center investigation. The study flowchart is shown in Figure 1.

### 2.2 Randomization and blinding

Randomization in this study is based on a computer-generated random number sequence. Participants will be assigned to intervention and standard care groups at a 1:1 ratio. Randomization will be carried out before patient enrollment so that each patient is assigned to the study’s respective group according to a pre-determined sequence, ensuring randomness and equity. Upon entry, every patient will be given an individual code that corresponds to the random number sequence, with continuous and smooth enrollment. Once randomized, patients will be transferred to an appropriate ward according to their assignment. Intervention group patients, for instance, will be admitted to Ward A, while control patients will be admitted to Ward B to avoid any possible cross-contamination between intervention strategies.

For the purpose of maintaining the confidentiality of the allocation sequence, an impartial team member will conduct the randomization process, who does not engage in participant recruitment nor gets involved in the allocation. This will prevent divulgence of the sequence in the course of the study. The team member will then communicate the allocation outcome to the intervention team, ensuring that neither the participant recruitment team nor the intervention team gains any prior knowledge of the allocation.

Lastly, due to the novelty of AI-HEALS intervention in this trial, total participant and therapist blinding is not possible. This limitation is bound to induce some biases, including performance bias due to participant unawareness of treatment assignment and detection bias due to therapists’ knowledge of the intervention adopted. To reduce these biases, we will implement a series of strategies to mitigate their effects on the study. First, confidentiality of all intervention and control group participants’ identities will be maintained until study completion. Second, a separate team member not involved in participant registration or intervention delivery will be responsible for conducting random allocation. An independent staff member will personally inform the intervention team of the allocation results, ensuring that the enrollment team remains blinded to group assignments. Third, intervention patients will be hospitalized in Ward A, with control patients in ward B, to avoid cross-contamination between study groups. Lastly, to further minimize bias factors, study data will be gathered by study assistants who remain blind to allocation. In the analysis process, data analysts will also remain unaware of group assignments. During the study, all data will be coded and stored in a password-protected, encrypted electronic database with access restricted to authorized study personnel only. Any physical documents will be kept in locked cabinets within secure research facilities. These measures ensure confidentiality and comply with institutional ethics requirements while preserving the integrity of the blinding process.

### 2.3 Study sample

All patients diagnosed with NSLBP are eligible to participate. Additionally, participants must meet the following criteria:

#### 2.3.1 Inclusion criteria

- According to the doctor’s clinical diagnosis, the participant meets the diagnostic criteria for nonspecific low back pain, which means there is no clear organic cause for the low back pain (1, 2).
- The duration of the low back pain exceeds 6 weeks, classifying it as chronic low back pain (3, 29).
- The participant owns a smartphone, is proficient in its use, and is willing to receive and participate in the intervention program through the WeChat platform.
- The participant is aged 18 or older.
- The participant is willing and able to sign an informed consent form and participate in the entire study period.

#### 2.3.2 Exclusion criteria

- Lack of basic reading and writing skills, unable to receive and understand health information via WeChat;
- Presence of severe cardiovascular, renal, or other major organ dysfunctions;
- Abnormal cognitive abilities or psychiatric conditions that preclude cooperation;
- Pregnancy or currently breastfeeding;
- Known severe allergies to any materials, devices, or components used in the intervention;
- Active substance abuse or dependence that may interfere with participation;
- Severe frailty, inability to independently use a smartphone, or inability to complete study follow-up;
- Currently participating in other similar research studies;
- Unwilling to cooperate or Other reasons that make participation in the trial inappropriate.

#### 2.3.3 Withdrawal criteria

- Participants who voluntarily request to withdraw from the study for personal reasons, such as scheduling conflicts or changes in interest;
- Occurrence of severe adverse events during the study, such as serious physical illnesses or acute events (e.g., heart attack, severe infection) that require interruption of the intervention;
- Changes in medical condition or other medical advice that necessitate altering the original treatment plan, potentially conflicting with the study intervention.

### 2.4 Sample size

G-Power 3.1.9.4 was used to calculate the sample size. The effect size was 0.68 (33), efficacy was 0.80, alpha was set at 0.05, and one-tailed tests were performed based on previous studies. The calculated required sample size was determined to be at least 58 (29 participants per group). We estimated an attrition rate of 20%, so a total sample of at least 74 participants (37 per group) was required.

### 2.5 Recruitment

It will be a study enrolling patients with NSLBP meeting inclusion and exclusion criteria at the designated hospital. Recruitment will be conducted by the medical staff at the hospital, which will identify such patients upon their intake and extend an invitation. Then, an initial face-to-face interaction with patients by the study team will take place, in which patients will be briefed on objectives, methods of the study, possible benefits, and potential risks. Invited patients will be brought to the hospital for a thorough health evaluation, involving a comprehensive history of their pain, as well as symptoms. Once study details have been clearly explained to the participant and their questions answered, they will be asked to sign an informed consent. Only after signing the document will patients be officially enrolled in the study.

### 2.6 Informed consent

The informed consent process will be carried out transparently and openly, with strict adherence to ethical principles. NSLBP patients meeting inclusion and exclusion criteria will be presented with a thorough written explanation of study aims, procedures, possible risks and rewards, and measures to protect patients’ privacy, including meticulous details of collecting, storing, and protecting their information. We will take care to keep all data securely on encrypted computers that only approved study members can access, only for the duration of the study, and will be properly destroyed upon study completion. Individuals will have ample time to read carefully through the consent form, ask questions, and provide consent voluntarily. Participants will be made aware clearly that they can withdraw from study participation at any time without adverse effects. Ongoing consulting contact information will be made available by the study team so that people can seek additional information or explanation at any time. Participants will receive any new information during the study that could influence participation continuation so that ongoing consent is maintained.

In addition, no penalties will be levied upon participants who choose to withdraw from the intervention arm. Patients who withdraw if intervention has already started cannot be reassigned to the control arm and will be counted as natural attrition cases. Where possible, semi-structured interviews with withdrawing participants will be undertaken to examine reasons for departure. Participants will be explicitly made aware of all accessible modes of follow-up in the event of withdrawal through an informed consent process, so that they understand all their rights and options well.

### 2.7 Intervention

#### 2.7.1 Control group

Control group members will be assigned to receive standard medical care by a multidisciplinary medical team. Healthcare services will consist of: (1) routine consultations with doctors for health check-ups and treatment regimen modifications; (2) follow-up visits after predetermined intervals to track disease progression; (3) thorough diagnostic evaluations to follow health indicators; (4) formal educational sessions addressing management of the condition, treatment compliance, and change of lifestyle; and (5) referrals to specialized medical care providers if clinically warranted. This approach maintains consistency with clinical practice guidelines and standard protocols for routine management of asthma.

#### 2.7.2 Intervention group

**Figure 2:**
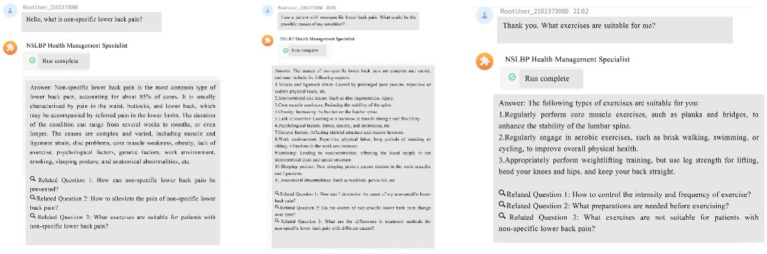
Artificial intelligence health education precision linkage system responses to non-specific lower back pain knowledge.

**Figure 3:**
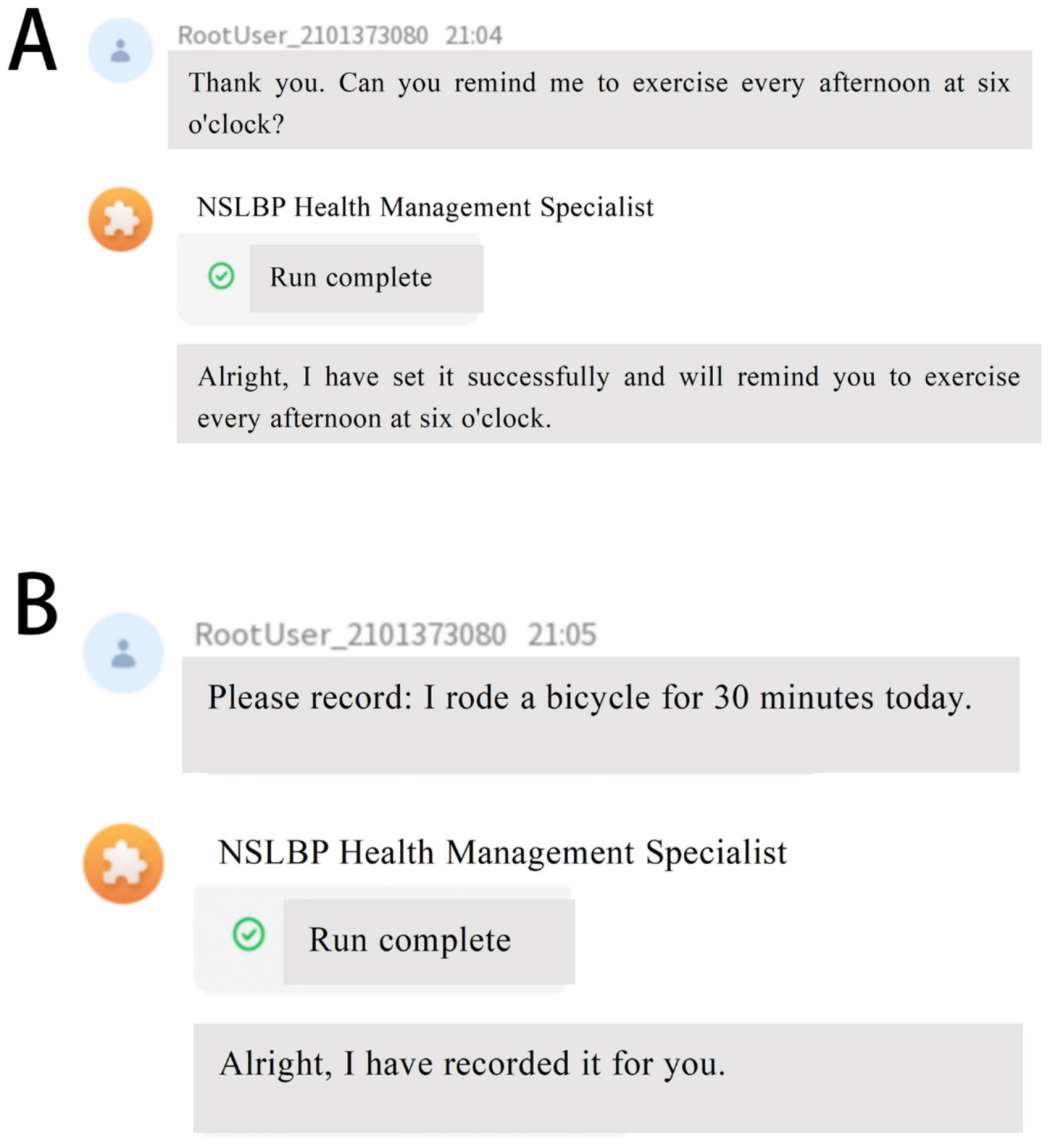
Reminder and Record Features of Artificial Intelligence Health Education Precision Linkage System. **(A)** Personalized reminder service to improve adherence to treatment and healthy behaviors; **(B)** Recording of lifestyle factors and physiological indices to facilitate long-term monitoring and self-management.

Based on earlier RCTs conducted on NSLBP (33), this trial uses a 3-month intervention duration. Throughout these 3 months, AI-HEALS will administer intervention to the intervention arm through the “NSLBP Health Management Expert” WeChat system. Intervention members include public health professionals, clinicians, clinical nurses, psychologists, and statisticians. Intervention program components consist of four major components:

·NSLBP Knowledge-Based Question-Answering System (KBQA): This system will enable NSLBP patients to better understand their illness. It works with a frequently asked questions robot (FAQrobot), which allows participants to access NSLBP knowledge efficiently and accurately. To make this possible, we will compile a robust and accurate NSLBP knowledge base encompassing various aspects such as general knowledge of the disease, causes, symptoms, treatment procedures, use of medications, day-to-day management, appropriate diet, and relevant physical exercise. We will incorporate this knowledge base into ByteDance’s large language model (LLM), which will enable the model to learn from the database and respond to questions professionally on the WeChat site. In addition, to make user interaction and engagement stronger, three relevant questions will be presented each time a user submits an inquiry, promoting in-depth exploration and interaction. To adaptively improve the knowledge base and respond to users’ demands, we will record participants’ query behaviors (e.g., system access times, frequencies) and uses in the system backend. By exploring these data, we can understand behavioral patterns based on which we improve and advance the knowledge base and question-answering system in such a way that information timeliness and accuracy are ensured. During the process of Q&A interaction, the system offers both text and voice inputs, which notably reduces the threshold for operation and allows broader accessibility (Figure 2).
·Recording Lifestyle Factors and Physiological Indices: AI-HEALS will assist NSLBP patients in documenting regularly their factors of lifestyle, namely week-by-week medications, foods consumed, exercise, etc. Participants can enter information in real-time, and it is possible for researchers to view recorded information through the backend interface. This will allow long-term tracking and monitoring of self-management practices. In order to make the data relevant and accurate, the team conducting the study will engage with the patients’ families at an early point in participation to design SMART (Specific, Measurable, Achievable, Relevant, Time-bound) plans. Data collection will be centered on these plans, with system-generated reminders being sent to all study participants annually on every Saturday to encourage them to upload rich information. This will enable a thorough examination of the intervention’s effects on patients’ everyday living in addition to ensuring that data captures the individual goals of each participant (Figure 3B).
- Personalized Reminder Service: To improve treatment adherence, the AI-HEALS system will offer personalized reminder services. Participants may easily configure reminders for different significant activities like drug intake and physical exercise. Once such reminders are configured, the system will provide reminders based on these specified times. This reminder feature will assist patients in strictly following medical advice, enhance long-term adherence to healthy behaviors, and ultimately aid them in managing their NSLBP (Figure 3A).
·Automated & Personalized NSLBP Education Articles for Patients: The system will also run an automated system for dispensing education articles. One to three education articles on topics related to managing pain will be sent every week to NSLBP patients. Examples of topics include exercise advice that helps patients with types of exercise that are appropriate and intensities of exercise, along with correct drug information like administration methods and possible side effects to watch for. Education articles will be exactly personalized and distributed depending on participant interaction patterns with the system like types of questions posed, frequency of visits, and areas of interest to address each NSLBP patient’s individual needs. In order to make information dissemination most effective, background data will be regularly checked by the research team, such as assessing read numbers, types of preferred papers, and time spent on each. Following these data, article content and frequency of distribution will be adjusted.

### 2.8 Overview of the artificial intelligence system

“Doubao-pro-32 k,” a Chinese-trained Transformer model optimized for medical knowledge application, was deployed in this study. Doubao-pro-32 k was trained by merging biomedical literature with pain-related clinical guidelines, after which it was fine-tuned on a 32 k context window to produce long-text health education content. Pre-training in general language understanding was carried out on the model before domain-specific fine-tuning to improve medical reasoning ability. Third-party performance benchmarking on the FlagEval evaluation platform^1^ revealed that “Doubao-pro-32 k” scored competitively with GPT-4o. Indeed, overall, “Doubao-pro-32 k” scored 77.75 points, beating GPT-4o’s 73.51 points. Its greatest strength lies in knowledge application (Doubao-pro-32 k: 91.14, GPT-4o: 86.71), an ability that is essential in producing evidence-based clinical information. On development, domain experts performed an initial review of the output from the model to confirm consistency with current medical knowledge and compliance with high levels of medical evidence-based research. Post-pilot study, a sustained validation program will be implemented, such that two experts review 10% of outputs month to month on a back-to-back basis and estimate rates of accuracy to keep system accuracy sustained.

Finally, under this study, maintaining participants’ privacy and securing data integrity in their interaction with the AI-HEALS system are top priority. Any patient data collected through the AI-HEALS system will be held with utmost confidentiality and only for purposes of conducting this study. Each member of our study team is bound by applicable privacy and confidentiality agreements, so both legally and ethically, they are bound to secure participants’ sensitive information. In addition, study participants are entitled to withdraw from participation unconditionally at any point in time without any grounds. This respect for participants’ autonomy guarantees that study members are not pushed to continue. Lastly, all adverse events resulting from intervention will be carefully documented and reported in accordance with local regulations and stipulated procedures.

### 2.9 Strategies to improve adherence to interventions

In order to ensure participant compliance, we will: (1) clearly discuss participant responsibilities upon participation in the observational period at study recruitment and upon signing of the informed consent. Simultaneously, we will work diligently to establish sound participant relationships to enable mutual trust. (2) Offer thorough education and counseling regarding disease management and prevention strategies throughout study. We will discuss disease characteristics, stress the need for strict treatment compliance, and provide tips for avoiding potential complications. (3) Weekly review by the research team of intervention group interaction with the system will ensure maximum participant engagement. Ongoing review will warrant early detection of any problem or disengagement, so that corrective actions can be initiated by the research team and participant engagement maintained.

### 2.10 Outcomes

#### 2.10.1 Primary outcomes

Changes in pain intensity from baseline to discharge, at the end of the intervention, and during follow-up periods at 3, 6, and 9 months post-intervention.

#### 2.10.2 Secondary outcomes

·Changes in self-management behaviors: Dietary habits, physical exercise, medication adherence, smoking management, alcohol management, sleep management, quality of life, and activities of daily living.
·Social cognition and psychology: NSLBP health literacy, self-efficacy, depression, anxiety, stress, and social support.
·Control of physiological indicators: blood pressure, height, weight.
·Additionally, recruitment rates, dropout rates, and reasons for dropout will be collected. To ensure data homogeneity, demographic characteristics and study outcomes of dropouts and participants with adversarial attitudes toward the trial will be compared during data analysis.

### 2.11 Variables measurement

Sociodemographic variables will be collected at baseline. Physical examinations and questionnaire surveys will be conducted at baseline, at discharge, at the end of the intervention, and at 3, 6, and 9 months post-intervention (Table 1).

**Table 1:** The schedule of enrolment, interventions, and assessments.

Study period	Recruitment	Intervention		Follow-up		
Timepoint	0	Discharge	3 Months	3 Months	6 Months	9 Months
Eligibility screen	√					
Informed consent	√					
Allocation	√					
Sociodemographic variables	√					
VAS	√	√	√	√	√	√
RMDQ	√	√	√	√	√	√
OPFEAS	√	√	√	√	√	√
PHQ4	√	√	√	√	√	√
PSSS-SF	√	√	√	√	√	√
NGSES-SF	√	√	√	√	√	√
EQ-5D-5L	√		√	√	√	√
IPAQ-SF	√		√	√	√	√
Smoking	√		√	√	√	√
Alcohol consumption	√		√	√	√	√
B-PSQI	√		√	√	√	√
SREBQ	√		√	√	√	√
FCS-SF	√					
eHEALS	√					

VAS, visual analog scale; RMDQ, The Roland-Morris Disability Questionnaire; OPFEAS, The Orthopedic Patient Functional Exercise Adherence Scale; PHQ-4, the abbreviated Patient Health Questionnaire-4; PSSS-SF, The Perceived Social Support Scale; NGSES-SF, The New General Self-Efficacy Scale-Short Form; EQ-5D-5L, EuroQol-5 Dimensions 5 Levels; IPAQ-SF, The International Physical Activity Questionnaire Short Form; B-PSQI, The new Brief Version of the Pittsburgh Sleep Quality Index-Short Form; FCS-SF, The Family Communication Scale-Short Form; SREBQ, The Self-Regulation of Eating Behavior Questionnaire; eHEALS, The eHealth Literacy Scale.

Sociodemographic variables to be collected include gender, age, marital status, location, average household size, educational level, and occupation, along with clinical information such as the duration of NSLBP and detailed medical history. Anthropometric variables: Each participant’s height, weight, waist circumference, and blood pressure will be measured twice using certified instruments. Height and weight will be measured using fully calibrated, certified electronic devices. Body Mass Index will be calculated by dividing weight (in kilograms) by height (in meters) squared. Waist circumference will be measured at the level of the navel using a tape measure. As for blood pressure, systolic and diastolic pressures will be measured using a validated automatic sphygmomanometer.

### 2.12 Questionnaires

·The Visual Analog Scale (VAS) for pain is one of the most commonly used standards for assessing pain. It employs a 10 cm line to represent the continuum of pain severity, marked with numerals from 0 to 10 to indicate varying degrees of pain. A score of 0 signifies “no pain” while 10 denotes “unbearable pain.” Participants are asked to mark a point on the line that corresponds to their subjective experience of pain, thereby quantifying the intensity of their pain (38).
·The Roland-Morris Disability Questionnaire (RMDQ) is a scale used to assess functional disability in patients with lower back pain (39). It consists of 24 items, each related to limitations in daily activities associated with lower back pain. Patients respond with “yes” or “no” to each item based on their condition to assess the impact of lower back pain on their daily activities. The scoring system is as follows: if a patient indicates that they are unable to perform an activity due to back pain, the item is scored as “yes” with 1 point; if the patient can perform the activity, the item is scored as “no” with 0 points. The total score is the sum of all item scores, ranging from 0 (no disability) to 24 (maximum disability). The scale has demonstrated high internal consistency, with Cronbach’s *α* values ranging from 0.84 to 0.96 (39).
·The Orthopedic Patient Functional Exercise Adherence Scale (OPFEAS) is designed to evaluate the adherence of orthopedic patients to functional exercises during treatment and rehabilitation (40). This scale includes 3 dimensions and 15 items that cover exercise adherence related to physical, psychological, and proactive learning aspects. It uses a 5-point Likert scale, where 1 indicates “not at all able” and 5 indicates “completely able,” with higher scores reflecting higher adherence to functional exercises. It has demonstrated excellent internal consistency, with a Cronbach’s *α* of 0.930.
·Depression and anxiety will be assessed using the abbreviated Patient Health Questionnaire-4 (PHQ-4), a brief self-report tool designed to evaluate symptoms of depression and anxiety over the past 2 weeks (41). The scale consists of four items, with the first two items related to depression and the last two related to anxiety. Each item is scored on a 4-point scale, with scoring options of “Not at all,” “Several days,” “More than half the days,” and “Nearly every day,” corresponding to scores of 0, 1, 2, and 3, respectively. The total score ranges from 0 to 12 points, with a Cronbach’s alpha of 0.833 (42).
·The Chinese version of Perceived Social Support Scale PSSS-SF is designed to assess an individual’s perception of social support (43). The scale comprises three items across three dimensions: family support (item 1), friend support (item 2), and other support (item 3). It utilizes a 7-point Likert scale for scoring, ranging from 1 (strongly disagree) to 7 (strongly agree), with total scores ranging from 3 to 21. Higher scores indicate a higher level of perceived social support, and the scale has demonstrated good internal consistency, with a Cronbach’s *α* of 0.84 (44).
·The Chinese version of New General Self-Efficacy Scale-Short Form (NGSES-SF) is used to evaluate an individual’s confidence and belief in their ability to successfully complete tasks or handle challenges (45). This scale includes three items across three dimensions: level or degree of self-efficacy (item 1), intensity (item 2), and generality (item 3), using a 5-point Likert scale for scoring, ranging from 1 (strongly disagree) to 5 (strongly agree), with total scores ranging from 3 to 15. Higher scores reflect a higher level of individual self-efficacy, and the scale has demonstrated good internal consistency, with a Cronbach’s *α* of 0.876 (45).
·The Chinese version of Quality of life will be assessed using the EQ-5D-5L (EuroQol-5 Dimensions 5 Levels). The EQ-5D-5L encompasses five dimensions: Mobility, Self-care, Usual activities, Pain/Discomfort, and Anxiety/Depression. Individuals select the appropriate level for each dimension based on their personal health status, forming a five-dimensional profile ranging from no problems to extreme problems (46). In addition to the descriptive system, the EQ-5D includes a VAS, allowing individuals to subjectively rate their current health status on a scale from 0 to 100. This score provides a continuous variable for measuring an individual’s overall quality of life. In seven studies involving cancer patients, the EQ-5D demonstrated high validity and reliability, with Cronbach’s alpha values ranging from 0.72 to 0.90 (47–53).
·The Chinese version of International Physical Activity Questionnaire Short Form (IPAQ-SF) consists of seven items designed to assess physical activity among adults aged 15 to 69 over the past 7 days, including vigorous, moderate, and walking activities (54). Participants are required to report the frequency and duration of each type of activity, as well as sedentary time during weekdays. Scoring is conducted by converting activity duration into metabolic equivalent of task (MET) minutes per week, with different activities assigned varying MET values, thereby quantifying an individual’s total physical activity level. This instrument is extensively utilized in public health research to evaluate population-level physical activity levels and their associated health impacts. It has demonstrated acceptable reliability, with an intraclass correlation coefficient (ICC) of 0.79 and a coefficient of variation (CV) of 26% (55).
·Smoking behavior is measured using a self-constructed questionnaire consisting of four multiple-choice questions that assess whether an individual smokes, the number of years since quitting, the number of cigarettes smoked per day, and the duration since starting smoking (56). Respondents will be asked, “Do you have a habit of smoking?” The answer options are: (1) Yes, I smoke regular cigarettes; (2) Yes, I vape; (3) Yes, I use both; (4) I used to smoke (but have quit); (5) No. Based on the responses, smoking status is categorized into two groups: (1) Smoker: currently smokes; (2) Non-smoker: never smoked or has quit smoking.
·Alcohol consumption is measured using a self-constructed questionnaire with seven multiple-choice questions that assess whether an individual drinks alcohol, age at the start of drinking, age at cessation of drinking, type of alcohol consumed, amount of alcohol consumed per day, amount of alcohol consumed per day before quitting, and anxiety about quitting alcohol. Respondents will be asked, “Do you currently or have you in the past consumed alcohol?” The answer options are: (1) I never drink; (2) I always drink; (3) I used to drink but now I do not; (4) I did not drink in the past but I do now.
·The Chinese version of new Brief Version of the Pittsburgh Sleep Quality Index-Short Form (B-PSQI) is a condensed version of the Pittsburgh Sleep Quality Index, used to measure an individual’s sleep quality (57). The scale consists of 6 items across 5 dimensions, specifically measuring sleep efficiency (items 1 and 2), sleep onset time (item 3), sleep duration (item 4), sleep disturbances (item 5), and overall sleep quality (item 6). Bedtime and wake-up time are used to calculate sleep efficiency, with a total score ranging from 0 to 15; higher scores indicate poorer sleep quality. The scale has demonstrated acceptable internal consistency, with a Cronbach’s *α* of 0.79 (57).
·The Chinese version of Family Communication Scale-Short Form (FCS-SF) is used to measure communication abilities within the family system (58). This scale consists of 4 items across a single dimension and utilizes a 5-point Likert scale, with scoring options ranging from 1 (strongly disagree) to 5 (strongly agree). Total scores range from 5 to 20, where higher scores indicate a higher level of family communication. The scale has demonstrated excellent internal consistency, with a Cronbach’s *α* of 0.91 (58).
·The Chinese version of Self-Regulation of Eating Behavior Questionnaire (SREBQ) is used to assess an individual’s ability to regulate their eating behaviors (59). This scale consists of 5 items across a single dimension, using a 5-point Likert scale with scores ranging from 1 (never) to 5 (always). Total scores range from 5 to 25, with higher scores indicating greater ability in regulating one’s dietary habits. The scale has demonstrated acceptable internal consistency, with a Cronbach’s α of 0.75 (59).
·The Chinese version of eHealth Literacy Scale (eHEALS) is designed to comprehensively evaluate an individual’s knowledge, ease of use, and perceived skills in searching, evaluating, and applying electronic health information to solving real health problems (60). This scale includes 5 items across a single dimension, utilizing a 5-point Likert scale, with scoring options ranging from 1 (strongly disagree) to 5 (strongly agree). Total scores range from 5 to 25, with higher scores indicating a higher level of personal eHealth literacy. It has demonstrated excellent internal consistency, with a Cronbach’s *α* of 0.913 (61).

### 2.13 Statistical analysis

In this study, quantitative data will be collected through questionnaires and stored in Microsoft Excel format. Data analysis will follow the intention-to-treat principle. For continuous variables, results will be presented as mean ± standard deviation or as median with interquartile range (P25, P75), depending on the normality of the distribution, which will be assessed using the Kolmogorov–Smirnov test. Appropriate statistical methods, such as Student’s t-test, one-way ANOVA, or non-parametric tests, will be used for group comparisons. Categorical variables will be expressed as counts and percentages (n, %) and analyzed using the chi-square test.

To explore the relationships between behavioral patterns and mobile health application use, linear regression, logistic regression, and structural equation modeling will be applied to cross-sectional data. For longitudinal analyses, generalized linear mixed models will be used to assess the impact of AI-HEALS interventions on behavior change and NSLBP management over time. Subgroup analyses will be conducted based on patients’ levels of family communication and eHealth literacy to refine and contextualize the findings.

Missing data will be addressed using multiple imputation by chained equations (MICE), with 50 iterations to ensure convergence. Predictive mean matching (PMM) with five nearest neighbors will be employed, generating 20 imputed datasets. All statistical tests will be two-sided, with a significance level set at 0.05. Data will be analyzed using IBM SPSS Statistics 24.0 (SPSS Inc., Chicago, IL, United States), Stata 14.0 (StataCorp, College Station, TX, United States), and Mplus 7.4 (Muthén & Muthén, Los Angeles, CA, United States).

Upon completion of the intervention and at 3, 6, and 9 months post-intervention, structured interviews will be conducted with participants to gather feedback. The structured interview guide will consist of approximately 8–10 open-ended questions covering four main areas: (1) perceived attractiveness and acceptability of the program, (2) usability and ease of engagement, (3) motivational and challenging factors for sustaining self-management behaviors, and (4) suggestions for further development of AI-HEALS. These interviews aim to provide deeper insights into the factors motivating participation and behavioral change. Participants will be invited to engage in approximately 30-min telephone or face-to-face in-depth interviews. Selection of participants will consider sociodemographic variables such as gender, age, location, education level, and pain intensity. Recruitment will continue until thematic saturation is achieved. Audio recordings of interviews will be transcribed verbatim, anonymized, and analyzed, with data coding and management conducted in NVivo (Version 12, QSR International, Doncaster, Australia). Both qualitative and quantitative data will be integrated to better interpret the results across multiple phases of the trial.

## 3 Study management

There will be a minimum of two members of the Ethics Committee of West China Jintang Hospital, Sichuan University with no conflict of interest with the study that will make up the Data Monitoring Committee (DMC). The DMC will be responsible for performing routine monitoring and review of studies and reporting to the Ethics Committee to ensure independence from the principal investigator. The DMC can suspend or end the study if they detect any deviation from the approved study protocol or unauthorized modifications in oversight. Every 6 months, reviews will be carried out by the Ethics Committee to check progress against the protocol established. Before trial commencement, the research team will meet with the principal investigator weekly to confirm adherence to protocol standards. Daily communications between the principal investigator and trial manager, with respect to protocol compliance, will be ensured by the trial team, by conducting meetings on a monthly basis during data collection.

Throughout the study, the research team is expected to immediately inform the Ethics Committee in case of any serious adverse event (SAE) or adverse event (AE). If a participant suffers an SAE or AE, regardless of whether it is study-related, the researchers must inform the research team in 24 h and stop the trial. Researchers should stop the trial instantaneously in case of severe errors in programing in the artificial intelligence system or severe issues that are noticed during interaction with users. The research team will then fix the technology and enhance the user interface of the AI system. Once the issues are resolved and checked, the study can be reconstructed and resumed for the sake of participant safety and study validity.

Prior to conducting the study, training sessions and evaluations will be conducted by specialists so that all members of the team are familiar with procedures and technical aspects. To avoid missing follow-ups, we will keep a record of all study participants and link each participant with a distinctive identification number. Further, to make intervention and comparison with the control group consistent, all times for assessments will be the same, and there will be use of similar validated questionnaires. We will have a double data entry and verification process to assure accuracy of the data. Additionally, we will consult statistician experts to determine the most suitable methodologies to use in the study. If we encounter an abnormal data point, we will review the original questionnaires to ensure the accuracy of the data. We will only use data for final analysis after ensuring their absolute accuracy.

The chief researcher will periodically review the work of the research team to ensure adherence to the research protocol and proper data collection procedures. If the research team fails to comply with surveying or follow-up procedures, the chief researcher may take corrective measures, including pausing the trial or altering the randomization scheme. In the event of an issue with questionnaire design or data recording errors, the research team will temporarily halt data collection to address the problem. Any revisions to the questionnaire will be limited to correcting formatting issues, typographical errors, or clarifying wording without altering the underlying constructs or scoring, in order to preserve measurement equivalence. If a substantive change to the questionnaire is deemed necessary, the same revised version will be administered to all affected participants to avoid mixing responses from different versions. Before redistribution, the revised questionnaire will undergo expert review and pilot testing to confirm content equivalence and internal reliability. Once validated, the revised questionnaire will be redistributed to ensure uniformity and reliability of data collection. A data administrator will oversee data management, while a trial administrator will monitor data recording, address issues related to cloud storage and data access, and perform regular checks to confirm that all information is accurate, trustworthy, and compliant with the research protocol.

Unblinding is permitted under this study under special conditions, such as medical emergencies like a serious adverse event or a need for emergency treatment in which knowledge of treatment assignments is urgently required. Participants or their health care providers may also request unblinding after study completion for legitimate medical reasons. To follow these steps for each unblinding request: A written request from the principal investigator or a clinical oversight staff member initiates unblinding. A review panel comprised of clinicians examines each unblinding request for eligibility. Upon approval, members of the study team will disclose group assignments according to the designated protocol in a way that does not pose a threat to study integrity.

All proposed changes to the study will be first brought before the Steering Committee. Once approved, revised content will be submitted to the Hospital Ethics Committee and research department for review and sign-off. Upon approval, there will be a formal document detailing changes made available to all stakeholders, with an electronic copy if required. All changes to the protocol will be recorded by the trial coordinator and signed by the principal investigator. This record will be stored with other trial documents. Once approved by the Ethics Committee, research department, and Ethics Committee, the trial coordinator will modify the clinical trial application.

## 4 Dissemination plans

A summary of the RCT findings will be prepared by our research team to be distributed via email to persons who indicated an interest in seeing their results on their consent forms. The results of the study will be disseminated through publication in national and international peer-review journals, and presentation at national and international conferences. The results of the study will also be made available to other hospitals for dissemination to their medical staff upon request. The results will be made available upon request.

## 5 Discussion

In this study protocol, we aim to rigorously assess the effects of AI-HEALS intervention on patients’ self-management capacity for NSLBP using an RCT. From these studies, we seek to identify the efficacy of the AI-HEALS intervention in enhancing pain management, promoting self-management activities, and enhancing social cognition and psychological status. While changes in pain intensity are considered the main outcome, changes in dietary behaviors, physical exercise frequency, drug regimen adherence, social cognition, and psychological aspects like health literacy for NSLBP, self-efficacy, depression, anxiety, and stress levels are considered secondary outcomes. We will also examine changes in these aspects. The intervention components in AI-HEALS involve a large language model KBQA built upon a knowledge base of our own design, physiological indicator tracking, reminder services with schedules, and tailored health management information, all with an aim to change patient behaviors favorably and health status.

According to systematic reviews from the past 5 years, numerous RCTs have explored the effects of mobile health interventions on pain patients (29, 31, 32). However, these interventions typically rely on traditional methods such as text messages, phone calls, or mobile social platforms (25, 27, 35). Compared to these, interventions combining large language models and mobile social platforms are relatively rare in the existing literature. Furthermore, although many studies focus on chronic pain, back pain, or musculoskeletal pain, specific research on NSLBP is relatively limited (22, 39, 59). Previous studies have primarily focused on outcomes such as pain and quality of life, often overlooking a systematic assessment of patients’ pain, physiological, psychological, and behavioral indicators (17, 37, 41). Thus, this study is intended to address this void by leveraging contemporary large language model technology, as well as mobile social media, in promoting an all-encompassing health management program that not only targets relieving pain but also encompasses an overall evaluation of patients’ physiological, psychological, and behavioral adjustments. Such an approach can provide better insights to treat NSLBP and improve prospects for mobile health intervention in managing chronic illnesses.

## Trial status

The study began in April 2025 and is expected to conclude in December 2026. This research protocol is the first version and was registered with the China Clinical Trial Registration Center on October 12, 2024 (Registration Number: CHICTR2400090707).

## Footnotes

^1^ https://flageval.baai.ac.cn

## References

[1] Bardin LD King P Maher CG. Diagnostic triage for low back pain: a practical approach for primary care. Med J Aust. (2017) 206:268–73. doi: 10.5694/mja16.00828, PMID: 28359011

[2] Tagliaferri SD Ng SK Fitzgibbon BM Owen PJ Miller CT Bowe SJ . Relative contributions of the nervous system, spinal tissue and psychosocial health to non-specific low back pain: multivariate meta-analysis. Eur J Pain. (2022) 26:578–99. doi: 10.1002/ejp.1883, PMID: 34748265

[3] Wirth B Schweinhardt P. Personalized assessment and management of non-specific low back pain. Eur J Pain. (2024) 28:181–98. doi: 10.1002/ejp.2190, PMID: 37874300

[4] Mattiuzzi C Lippi G Bovo C. Current epidemiology of low back pain. J Hospital Manag Health Policy. (2020) 4:4. doi: 10.21037/jhmhp-20-17, PMID: 40809281

[5] Sribastav SS Peiheng H Jun L Zemin L Fuxin W Jianru W . Interplay among pain intensity, sleep disturbance and emotion in patients with non-specific low back pain. Peer J. (2017) 5:e3282. doi: 10.7717/peerj.3282, PMID: 28533953 PMC5436560

[6] Du S Hu Y Bai Y Hu L Dong J Jin S . Emotional distress correlates among patients with chronic nonspecific low back pain: a hierarchical linear regression analysis. Pain Pract. (2019) 19:510–21. doi: 10.1111/papr.12772, PMID: 30739397

[7] Marin TJ Van Eerd D Irvin E Couban R Koes BW Malmivaara A . Multidisciplinary biopsychosocial rehabilitation for subacute low back pain. Cochrane Database Syst Rev. (2017) 6:CD002193. doi: 10.1002/14651858.CD002193.pub2, PMID: 28656659 PMC6481490

[8] Jiang Y Wang Y Wang R Zhang X Wang X. Differences in pain, disability, and psychological function in low back pain patients with and without anxiety. Front Physiol. (2022) 13:906461. doi: 10.3389/fphys.2022.906461, PMID: 36406992 PMC9669742

[9] Merminod G Weber O Semlali I Terrier A Decosterd I Rubli Truchard E . Talking about chronic pain in family settings: a glimpse of older persons’ everyday realities. BMC Geriatr. (2022) 22:358. doi: 10.1186/s12877-022-03058-8, PMID: 35461217 PMC9034600

[10] Saito T Shibata M Hirabayashi N Honda T Morisaki Y Anno K . Family dysfunction is associated with chronic pain in a community-dwelling Japanese population: the Hisayama study. Eur J Pain. (2023) 27:518–29. doi: 10.1002/ejp.2076, PMID: 36585949

[11] Adnan R Van Oosterwijck J Danneels L Willems T Meeus M Crombez G . Differences in psychological factors, disability and fatigue according to the grade of chronification in non-specific low back pain patients: a cross-sectional study. J Back Musculoskelet Rehabil. (2020) 33:919–30. doi: 10.3233/BMR-191548, PMID: 33016899

[12] Wu A March L Zheng X Huang J Wang X Zhao J . Global low back pain prevalence and years lived with disability from 1990 to 2017: estimates from the global burden of disease study 2017. Annals Translational Med. (2020) 8:299. doi: 10.21037/atm.2020.02.175, PMID: 32355743 PMC7186678

[13] Hayden JA Ellis J Ogilvie R Stewart SA Bagg MK Stanojevic S . Some types of exercise are more effective than others in people with chronic low back pain: a network meta-analysis. Aust J Phys. (2021) 67:252–62. doi: 10.1016/j.jphys.2021.09.004, PMID: 34538747

[14] Yang Y Lai X Li C Yang Y Gu S Hou W . Focus on the impact of social factors and lifestyle on the disease burden of low back pain: findings from the global burden of disease study 2019. BMC Musculoskelet Disord. (2023) 24:679. doi: 10.1186/s12891-023-06772-5, PMID: 37633880 PMC10464198

[15] Li Y Zou C Guo W Han F Fan T Zang L . Global burden of low back pain and its attributable risk factors from 1990 to 2021: a comprehensive analysis from the global burden of disease study 2021. Front Public Health. (2024) 12:1480779. doi: 10.3389/fpubh.2024.1480779, PMID: 39606072 PMC11598917

[16] Oliveira CB Maher CG Pinto RZ Traeger AC Lin C-WC Chenot J-F . Clinical practice guidelines for the management of non-specific low back pain in primary care: an updated overview. Eur Spine J. (2018) 27:2791–803. doi: 10.1007/s00586-018-5673-2, PMID: 29971708

[17] Schreijenberg M Koes BW Lin C-WC. Guideline recommendations on the pharmacological management of non-specific low back pain in primary care – is there a need to change? Expert Rev Clin Pharmacol. (2019) 12:145–57. doi: 10.1080/17512433.2019.1565992, PMID: 30618319

[18] Koes BW Backes D Bindels PJ. Pharmacotherapy for chronic non-specific low back pain: current and future options. Expert Opin Pharmacother. (2018) 19:537–45. doi: 10.1080/14656566.2018.1454430, PMID: 29578822

[19] Lin H Wang X Feng Y Liu X Liu L Zhu K . Acupuncture versus Oral medications for acute/subacute non-specific low Back pain: a systematic review and Meta-analysis. Curr Pain Headache Rep. (2024) 28:489–500. doi: 10.1007/s11916-023-01201-7, PMID: 38190024 PMC11156714

[20] Zhang Y Tang S Chen G Liu Y. Chinese massage combined with core stability exercises for nonspecific low back pain: a randomized controlled trial. Complement Ther Med. (2015) 23:1–6. doi: 10.1016/j.ctim.2014.12.005, PMID: 25637146

[21] Ahmed NZ Anwar N Begum S Parvez A Ezhil R Anjum N. Effect of Ḥijāma (wet cupping), Dalk (massage) and Bukhūr (medicated steam) in amelioration of Waja al-Zahr (non-specific low back pain)–an open prospective clinical trial. J Complementary Integrative Med. (2022) 19:1025–32. doi: 10.1515/jcim-2021-0099, PMID: 34265876

[22] Veillon J Preuilh A Wormser J. Cognitive behavioural interventions led by a physiotherapist in chronic non-specific low back pain: a systematic review and meta-analysis. J Bodyw Mov Ther. (2024) 39:635–44. doi: 10.1016/j.jbmt.2024.03.057, PMID: 38876697

[23] Hochheim M Ramm P Amelung V. The effectiveness of low-dosed outpatient biopsychosocial interventions compared to active physical interventions on pain and disability in adults with nonspecific chronic low back pain: a systematic review with meta-analysis. Pain Pract. (2023) 23:409–36. doi: 10.1111/papr.13198, PMID: 36565010

[24] Young JJ Kongsted A Hartvigsen J Ammendolia C Jensen RK. Similar improvements in patient-reported outcomes for non-specific low back pain patients with and without lumbar spinal stenosis symptoms following a structured education and exercise therapy program. BMC Musculoskelet Disord. (2023) 24:839. doi: 10.1186/s12891-023-06950-5, PMID: 37880624 PMC10599001

[25] Lytras D Iakovidis P Sykaras E Kottaras A Kasimis K Myrogiannis I . Effects of a tailored mat-Pilates exercise program for older adults on pain, functioning, and balance in women with chronic non-specific low back pain: a randomized controlled trial. Aging Clin Exp Res. (2023) 35:3059–71. doi: 10.1007/s40520-023-02604-7, PMID: 37934400

[26] Fernández-Rodríguez R Álvarez-Bueno C Cavero-Redondo I Torres-Costoso A Pozuelo-Carrascosa DP Reina-Gutiérrez S . Best exercise options for reducing pain and disability in adults with chronic low back pain: pilates, strength, core-based, and mind-body. A network meta-analysis. J Orthop Sports Phys Ther. (2022) 52:505–21. doi: 10.2519/jospt.2022.10671, PMID: 35722759

[27] Union IT. Measuring the information society report. (2021). Available online at: https://www.itu.int/en/ITU-D/Statistics/Documents/publications/mis2014/MIS2014_without_Annex_4.pdf (Accessed on August 31, 2025).

[28] Union IT. Digital Trends in Europe 2021. Society Report Available online at: https://wwwituint/pub/D-IND (accessed on 6 March 2021). (2021).

[29] Siddi F Amedume A Boaro A Shah A Abunimer AM Bain PA . Mobile health and neurocognitive domains evaluation through smartphones: a meta-analysis. Comput Methods Prog Biomed. (2021) 212:106484. doi: 10.1016/j.cmpb.2021.106484, PMID: 34736169

[30] Li R Liang N Bu F Hesketh T. The effectiveness of self-Management of Hypertension in adults using Mobile health: systematic review and Meta-analysis. JMIR Mhealth Uhealth. (2020) 8:e17776. doi: 10.2196/17776, PMID: 32217503 PMC7148553

[31] McKay FH Cheng C Wright A Shill J Stephens H Uccellini M. Evaluating mobile phone applications for health behaviour change: a systematic review. J Telemed Telecare. (2018) 24:22–30. doi: 10.1177/1357633X16673538, PMID: 27760883

[32] Mikulski BS Bellei EA Biduski D De Marchi ACB. Mobile health applications and medication adherence of patients with hypertension: a systematic review and meta-analysis. Am J Prev Med. (2022) 62:626–34. doi: 10.1016/j.amepre.2021.11.003, PMID: 34963562

[33] Piette JD Newman S Krein SL Marinec N Chen J Williams DA . Patient-centered pain care using artificial intelligence and mobile health tools: a randomized comparative effectiveness trial. JAMA Intern Med. (2022) 182:975–83. doi: 10.1001/jamainternmed.2022.3178, PMID: 35939288 PMC9361183

[34] Fatoye F Gebrye T Fatoye C Mbada CE Olaoye MI Odole AC . The clinical and cost-effectiveness of telerehabilitation for people with nonspecific chronic low back pain: randomized controlled trial. JMIR Mhealth Uhealth. (2020) 8:e15375. doi: 10.2196/15375, PMID: 32357128 PMC7381065

[35] Zhang C-Q Zhang R Schwarzer R Hagger MS. A meta-analysis of the health action process approach. Health Psychol. (2019) 38:623–37. doi: 10.1037/hea0000728, PMID: 30973747

[36] Jiang Y Sun X Jiang M Min H Wang J Fu X . Impact of a mobile health intervention based on multi-theory model of health behavior change on self-management in patients with differentiated thyroid cancer: protocol for a randomized controlled trial. Front Public Health. (2024) 12:1327442. doi: 10.3389/fpubh.2024.1327442, PMID: 38282759 PMC10808536

[37] Sharma M Awan A Kapukotuwa S. Mini review: possible role of the multi-theory model of health behavior change in designing substance use prevention and treatment interventions. Front Public Health. (2024) 12:1298614. doi: 10.3389/fpubh.2024.1298614, PMID: 38496384 PMC10940529

[38] Gallagher EJ Liebman M Bijur PE. Prospective validation of clinically important changes in pain severity measured on a visual analog scale. Ann Emerg Med. (2001) 38:633–8. doi: 10.1067/mem.2001.118863, PMID: 11719741

[39] Stevens ML Lin CC Maher CG. The Roland Morris disability questionnaire. Aust J Phys. (2016) 62:116. doi: 10.1016/j.jphys.2015.10.003, PMID: 26687949

[40] Tan YY He H Yang XX Li XS Mi JX. Development and reliability and validity test of functional exercise adherence scale for orthopedic patients. China Nursing Manag. (2019) 19:34–9. doi: 10.3969/j.issn.1672-1756.2019.11.007

[41] Kroenke K Spitzer RL Williams JB Löwe B. An ultra-brief screening scale for anxiety and depression: the PHQ–4. Psychosomatics. (2009) 50:613–21. doi: 10.1176/appi.psy.50.6.613, PMID: 19996233

[42] Jie Q Minmin J Chen C Yujiao C Dehua Y Chunbo L. A study of the reliability and validity of the ultra-simple depression and anxiety screening scale in a community-based outpatient clinic. Internal Med Theory Prac. (2021) 16:116–20. doi: 10.16138/j.1673-6087.2021.02.010

[43] Blumenthal JA Burg MM Barefoot J Williams RB Haney T Zimet G. Social support, type a behavior, and coronary artery disease. Psychosom Med. (1987) 49:331–40. 3615762 10.1097/00006842-198707000-00002

[44] Wu Y Tang J Du Z Zhang X Wang F Sun X. Development of a short version of the perceived social support scale: based on classical test theory and ant colony optimization. BMC Public Health. (2022) 25:232. doi: 10.1186/s12889-025-21399-y PMC11745013 39833852

[45] Wang F Chen K Du Z Wu Y Tang J Sun X. Reliability and validity analysis and Mokken model of New General Self-Efficacy Scale-Short Form (NGSES-SF). PsyArXiv. Preprint posted online (2022). doi: 10.31234/osf.io/r7aj3

[46] Kaambwa B Ratcliffe J. Predicting euro QoL 5 dimensions 5 levels (EQ-5D-5L) utilities from older people’s quality of life brief questionnaire (OPQoL-brief) scores. Patient-Patient-Centered Outcomes Res. (2018) 11:39–54. doi: 10.1007/s40271-017-0259-3, PMID: 28623629

[47] Xu RH Cheung AW Wong EL. The relationship between shared decision-making and health-related quality of life among patients in Hong Kong SAR, China. Int J Qual Health Care. (2017) 29:534–40. doi: 10.1093/intqhc/mzx067, PMID: 28586442

[48] Huang W Yang J Liu Y Liu C Zhang X Fu W . Assessing health-related quality of life of patients with colorectal cancer using EQ-5D-5L: a cross-sectional study in Heilongjiang of China. BMJ Open. (2018) 8:e022711. doi: 10.1136/bmjopen-2018-022711, PMID: 30530472 PMC6286482

[49] Gavin AT Donnelly D Donnelly C Drummond FJ Morgan E Gormley GJ . Effect of investigation intensity and treatment differences on prostate cancer survivor's physical symptoms, psychological well-being and health-related quality of life: a two country cross-sectional study. BMJ Open. (2016) 6:e012952. doi: 10.1136/bmjopen-2016-012952, PMID: 27993906 PMC5168701

[50] Lloyd AJ Kerr C Penton J Knerer G. Health-related quality of life and health utilities in metastatic castrate-resistant prostate cancer: a survey capturing experiences from a diverse sample of UK patients. Value Health. (2015) 18:1152–7. doi: 10.1016/j.jval.2015.08.012, PMID: 26686802

[51] Philipp-Dormston W Müller K Novak B Strömer K Termeer C Hammann U . Patient-reported health outcomes in patients with non-melanoma skin cancer and actinic keratosis: results from a large-scale observational study analysing effects of diagnoses and disease progression. J Eur Acad Dermatol Venereol. (2018) 32:1138–46. doi: 10.1111/jdv.14703, PMID: 29150868 PMC6032898

[52] Noel CW Lee DJ Kong Q Xu W Simpson C Brown D . Comparison of health state utility measures in patients with head and neck cancer. JAMA Otolaryngol–Head & Neck Surg. (2015) 141:696–703. doi: 10.1001/jamaoto.2015.1314 26204439

[53] Mastboom MJ Planje R van de Sande MA. The patient perspective on the impact of tenosynovial giant cell tumors on daily living: crowdsourcing study on physical function and quality of life. Interactive J Medical Res. (2018) 7:e9325. doi: 10.2196/ijmr.9325 PMC5845102 29475829

[54] Craig CL Marshall AL Sjöström M Bauman AE Booth ML Ainsworth BE . International physical activity questionnaire: 12-country reliability and validity. Med Sci Sports Exerc. (2003) 35:1381–95. doi: 10.1249/01.MSS.0000078924.61453.FB, PMID: 12900694

[55] Macfarlane DJ Lee CC Ho EY Chan KL Chan DT. Reliability and validity of the Chinese version of IPAQ (short, last 7 days). J Sci Med Sport. (2007) 10:45–51. doi: 10.1016/j.jsams.2006.05.003, PMID: 16807105

[56] Chen J Luo M Gan L Li H Liu S Ren N . The association between smoking and family health with the mediation role of personality among Chinese people: nationwide cross-sectional study. BMC Psychiatry. (2024) 24:206. doi: 10.1186/s12888-024-05654-x, PMID: 38486183 PMC10941408

[57] Sancho-Domingo C Carballo JL Coloma-Carmona A Buysse DJ. Brief version of the Pittsburgh sleep quality index (B-PSQI) and measurement invariance across gender and age in a population-based sample. Psychol Assess. (2021) 33:111–21. doi: 10.1037/pas0000959, PMID: 33119375

[58] Guo N Ho HC Wang MP Lai AY Luk TT Viswanath K . Factor structure and psychometric properties of the family communication scale in the Chinese population. Front Psychol. (2021) 12:736514. doi: 10.3389/fpsyg.2021.736514, PMID: 34867617 PMC8632692

[59] Kliemann N Beeken RJ Wardle J Johnson F. Development and validation of the self-regulation of eating behaviour questionnaire for adults. Int J Behav Nutr Phys Act. (2016) 13:1–11. doi: 10.1186/s12966-016-0414-6, PMID: 27484457 PMC4969721

[60] Koo M Norman CD Hsiao-Mei C. Psychometric evaluation of a Chinese version of the eHealth literacy scale (eHEALS) in school age children. Int Electron J Health Educ. (2012) 15:29–36.

[61] Guo S Yu X Nie D Li X Wang L. Adaptation and evaluation of Chinese version of eHEALS and its usage among senior high school students. China J Health Educ. (2013) 29:106–8. doi: 10.16168/j.cnki.issn.1002-9982.2013.02.019

